# Hydration and Mechanical Properties of Blended Cement with Copper Slag Pretreated by Thermochemical Modification

**DOI:** 10.3390/ma15103477

**Published:** 2022-05-12

**Authors:** Daolin Wang, Qinli Zhang, Yan Feng, Qiusong Chen, Chongchun Xiao, Hongpeng Li, Yujing Xiang, Chongchong Qi

**Affiliations:** 1 School of Resource and Safety Engineering, Central South University, Changsha 410083, China; daolinw@csu.edu.cn (D.W.); zhangqinli@126.com (Q.Z.); qiusong.chen@csu.edu.cn (Q.C.); xiangyujing232@foxmail.com (Y.X.); 2 Feny Co., Ltd., Changsha 410083, China; xiaocc@csu.edu.cn; 3 Jiangxi Copper Group Yinshan Mining Co., Ltd., Dexing 334200, China; 13840452960@163.com; 4 State Key Laboratory of Safety and Health for Metal Mines, Maanshan 243000, China; chongchong.qi@csu.edu.cn

**Keywords:** granulated copper slag, chemical reconstruction, pozzolanic activity, hydration process, hydration product

## Abstract

The application of granulated copper slag (GCS) to partially replace cement is limited due to its low pozzolanic activity. In this paper, reconstituted granulated copper slag (RGCS) was obtained by adding alumina oxide (Al_2_O_3_) to liquid copper slag. Blended cement pastes were formulated by a partial substitute for ordinary Portland cement (OPC) with the RGCS (30 wt%). The pozzolanic activity, mechanical development, and the microstructure were characterized. The results show that 5–10 wt% Al_2_O_3_ contributes to the increase in magnetite precipitation in RGCS. The addition of Al_2_O_3_ alleviates the inhibition of C_3_S by RGCS and accelerates the dissociation of RGCS active molecules, thus increasing the exothermic rate and cumulative heat release of the blended cement pastes, which are the highest in the CSA10 paste with the highest Al_2_O_3_ content (10 wt%) in RGCS. The unconfined compressive strength (UCS) values of blended cement mortar with 10 wt% Al_2_O_3_ added to RGCS reach 27.3, 47.4, and 51.3 MPa after curing for 7, 28 and 90 d, respectively, which are the highest than other blended cement mortars, and even exceed that of OPC mortar at 90 d of curing. The pozzolanic activity of RGCS is enhanced with the increase in Al_2_O_3_ addition, as evidenced by more portlandite being consumed in the CSA10 paste, forming more C-S-H (II) gel with a higher Ca/Si ratio, and a more compact microstructure with fewer pores than other pastes. This work provided a novel, feasible, and clean way to enhance the pozzolanic activity of GCS when it was used as a supplementary cementitious material.

## 1. Introduction

Energy consumption, resource scarcity, and environmental pollution are three major challenges facing the world today. Cement is an important basic building material, and its production is accompanied by the consumption of resources and energy, as well as the emission of dust and harmful gases. The energy consumption and carbon emissions of cement production account for approximately 5.3% and 7% of the total global energy consumption and carbon emissions, respectively [1,2]. Furthermore, with the expansion of industry, especially in developing countries, the increase in industrial solid waste production will cause serious environmental problems. One current approach is to reduce cement consumption and industrial solid waste disposal by using industrial by-products with hydraulic or pozzolanic activity as supplementary cementitious materials (SCM) in cement products. However, the utilization of high-performance SCMs, such as ground granulated blast furnace slag, fly ash, and silica fume [3], is approaching saturation. Therefore, the utilization of non-ferrous smelting slags with low reactivity as SCM is attractive, with technological, economic, and environmental benefits.

Copper slag (CS) is an industrial solid waste produced in the copper smelting process. About 80% of copper is obtained by flotation, smelting, and refining [4]. A large quantity of CS is inevitably produced due to the low content of copper concentrate in the high-temperature smelting process. Approximately 2.2–3.0 tons of CS are generated for every ton of copper produced, and the global annual output of CS is about 40 million tons [5]. Granulated copper slag (GCS) exhibits pozzolanic activity due to its high content of glassy phase and silicon dioxide (SiO_2_). One current approach for the environmentally friendly treatment of GCS is to partially replace cement in concrete or paste backfilling materials. However, GCS is difficult to be directly used as SCM due to its low pozzolanic activity [6]. Commonly used methods to improve pozzolanic activity of materials include mechanical activation [7,8] and alkali activation [9]. Our previous research has confirmed that mechanical activation via vibratory milling up 3 h can effectively improve the pozzolanic activity of GCS, but with high-energy power consumption [10]. Alkali activation is mainly achieved through the chemical reaction between GCS and alkali activators. However, the main reason for the low pozzolanic activity of GCS is the high degree of network polymerization [11]. As a result, it is difficult for the above activation methods to fundamentally enhance the reactivity of GCS. During the metallurgical process, the temperature of liquid CS may reach 1250 °C [12] when it is discharged from the smelting furnace. Thus, a feasible way to modify the chemical and mineralogical compositions of GCS by adding appropriate regulators to liquid CS is worth investigation. Indeed, we have investigated the effect of CaO addition to liquid CS on the pozzolanic activity and glassy structure of GCS [13] with promising results, while alumina oxide (Al_2_O_3_), an amphoteric oxide, seems to have a more outstanding performance than CaO in lowering the liquid phase line temperature of slag [14] and improving the calcium aluminate phase (C_3_A) synthesis in ordinary Portland cement (OPC) [15].

Park et al. [16] found that the relationship between added Al_2_O_3_ content and slag viscosity is V-shaped in the metallurgical process. The mechanism of this action was predominantly related to the amphoteric behavior of Al_2_O_3_ [17]. Mihailova and Mehandjiev [18] used Fourier transform infrared spectroscopy to observe that, when less than 5 wt% of Al_2_O_3_ is added to CS, the resultant primary phase is forsterite. The asymmetric stretching band of [AlO_4_]^5−^ gradually occupies the dominant position and the characteristic diffraction peak of iron spinel appears when more than 10 wt% of Al_2_O_3_ is added. Kim et al. [19] also experimentally verified the effect of Al_2_O_3_ on the viscosity of CaO-SiO_2_-10 wt% MgO-Al_2_O_3_ slag. Additionally, Kim et al. found that an appropriate quantity of Al_2_O_3_ added helps to accelerate the dissociation of silicate network in GCS glassy phase [20]. However, the addition of Al_2_O_3_ into liquid CS on the pozzolanic activity of GCS has not been reported in the relevant studies.

Therefore, the present study investigates the pozzolanic activity of the restructured GCS (RGCS), which was obtained by adding Al_2_O_3_ into liquid CS followed by quenching with water. Physicochemical properties and mineral phase of RGCS were examined. The hydration heat and hydration products of blended cement paste samples with the RGCS were analyzed by isothermal calorimetry, X-ray diffraction (XRD), differential thermal analysis (DTA)/thermogravimetric analysis (TGA), and Fourier transform infrared spectroscopy (FTIR). The unconfined compressive strength (UCS) of blended cement mortar was testing to characterize mechanical behavior. The microstructures and structural compositions of paste samples were investigated using a scanning electron microscope (SEM) and an energy dispersive spectrometer (EDS). The results obtained from this study will provide theoretical support to improve the pozzlanic activity of non-ferrous smelting slag with a low-carbon and environmentally friendly method.

## 2. Materials and Methods

### 2.1. Materials

The original GCS, produced by melting, water quenching, granulation, drying, and ball milling, was obtained from the Rönnskär smelter of Boliden Mineral AB, Sweden. The modifying reagent was analytical pure reagent Al_2_O_3_, provided by Jiangxi Hengsheng New Material Co., Ltd in Jian, Jiangxi, China. Byggcement (CEM II/A-LL 42.5 R), was used as OPC for a control group, which was provided by a cement plant in Changsha, Hunan, China.

### 2.2. Samples Synthesis

**Figure 1:**
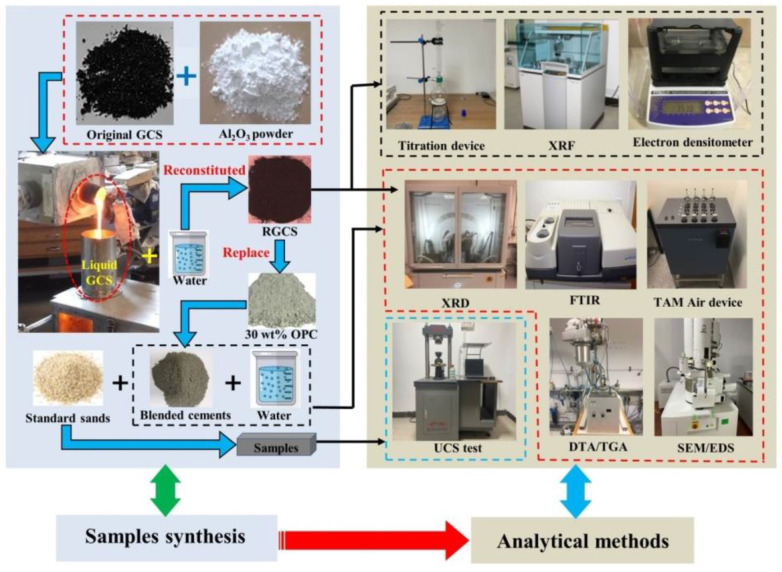
Sample preparation and synthesis procedure for experiments.

Given in Figure 1 is the synthesis procedure of samples preparation and the experiments.

#### 2.2.1. RGCS 

The RGCS was synthesized by adding different proportions of Al_2_O_3_ to liquid GCS. The specific methods and details of preparation have been described by previous studies [21]. Three different proportions of Al_2_O_3_ additions were selected to modify GCS according to the addition of Al_2_O_3_ in slag smelting [22]. Of these, the original GCS without Al_2_O_3_ (0 wt%) was coded CSA0. The mixture of GCS and Al_2_O_3_ containing 5 wt% Al_2_O_3_ was coded CSA05. Similarly, the mixture with 10 wt% Al_2_O_3_ was coded CSA10. 

#### 2.2.2. Blended Cement Paste

The three samples of RGCS were used to replace 30 wt% OPC to form GCS+OPC blended cements and were coded CSA0, CSA05, and CSA10. Then, 50 wt% paste was prepared from the three blended cements and OPC. The paste was poured into a cubic mold (4 × 4 × 4 cm) and cured in a curing box of constant temperature (25 °C, 90% humidity [23,24]) in which it solidified. The samples were dried in an oven at 50 °C for 3 d after 28 days of curing and then ground to stop their hydration reaction [25].

#### 2.2.3. Blended Cement Mortar

OPC and blended cement (70% OPC + 30% RGCS) were used with water and CEN Standard sand to form a homogeneous mortar with a cement-to-sand ratio of 1:3 and 50 wt% according to the Chinese standard GB/T 17671-1999 [26]. The mortar was then poured into a cubic mold (40 × 40 × 160 mm). The specific operation and maintenance requirements have been described by previous studies [27]. The mortar prepared using OPC was named P1 and used as the control group, while the mortars prepared with blended cement were named C0, C5, and C10 according to the content of Al_2_O_3_ in the RGCS.

### 2.3. Analytical Methods

#### 2.3.1. Physicochemical Properties and Mineral Phase Tests of RGCS

The compound composition and content of these samples were quantitatively described by XRF. The ferrous oxide (FeO) and iron oxide (Fe_2_O_3_) content in the RGCS were determined by titration. The density and BET surface area of RGCS were measured using the electron densitometer (Etnaln, ET-320R). The samples were scanned using XRD (Empyrean, PANalytical) to determine the mineral phase in the scanning range from 10° to 80°. In addition, the phase and its main components were qualitatively analyzed using the HighScore software [28].

#### 2.3.2. Pozzolanic Activity Test and Mineral Phase of Blended Cement

The early hydration heat release of blended cement paste within 120 h was continuously monitored by an isothermal conduction calorimeter (TAM Air device, TA Instruments) at 25 °C. The mineral phase of these blended cement samples at 28 d was analyzed by XRD in the scanning range of 10–70° to qualitatively describe the degree of formation of hydration products of blended cement. The analysis conditions were as described above. To further quantitatively describe pozzolanic activity of blended cement, after drying and grinding, the DTA and TGA were performed on the samples to determine the content of hydrated calcium silicate (C-S-H), calcium hydroxide (CH), and calcium carbonate (CaCO_3_). The STA 449C apparatus (Netzsch, Bavaria, Germany) was heated from room temperature (20 °C) to 900 °C at a cooling rate of 10 °C/min. Meanwhile, N_2_ (purity 99.996%) was continuously introduced at a flow rate of 100 mL/min to protect the samples during the test. The chemical bonding characteristics of hydration products were analyzed by FTIR (Nicolet Nexus 470 spectrometer) with KBr wafer in the wavenumber range 4000–400 cm^−1^.

#### 2.3.3. UCS Testing

The UCS test was conducted to obtain the macro cementation performance of blended cement [29]. Additionally, the UCS values measured by a SANS CDT1305 uniaxial electronic pressure tester at the curing ages of 7 d, 28 d, and 90 d [30]. To quantitatively describe the strength difference between the OPC and blended cement, 70% of the P1 UCS value was taken as the standard strength at each age. The ratio of 70% UCS value between the experimental group and the control group at the same age, called the UCS ratio, was obtained. Additionally, the ratio of the UCS values at different ages was defined as the UCS development rate to intuitively describe the development of UCS value of each mortar with age.

#### 2.3.4. Morphology and Structure Test of Blended Cement

The samples were evacuated and sprayed with gold by the JEC-3000FC automatic ion sputtering instrument to characterize the microstructure after curing for 28 d. Each sample was then cut into a cube of approximately 125 mm^3^ (5.0 × 5.0 × 5.0 mm [31]). Subsequently, the morphology and structure were observed by a JSM-IT500 SEM under an accelerating voltage of 20 kV and emission current of 1.0 nA. Further, the chemical composition of hydration products was analyzed using an X-ray energy spectrometer (EDS).

## 3. Results and Discussion

### 3.1. Physicochemical Properties 

Table 1 shows the physicochemical properties of RGCS and OPC. The Al_2_O_3_ accounted for approximately 3.5% of original GCS and increased significantly with the addition of Al_2_O_3_ in RGCS, while the content of most other oxides decreased. Convincing evidence for the fusion of Al_2_O_3_ and GCS in the molten state was provided. The BET surface area and density of RGCS were much larger than that of OPC, which slowly changed with the addition of Al_2_O_3_. This demonstrates that the effect of Al_2_O_3_ addition on the physical properties of RGCS is almost negligible.

### 3.2. Mineralogy of the RGCS 

**Figure 2:**
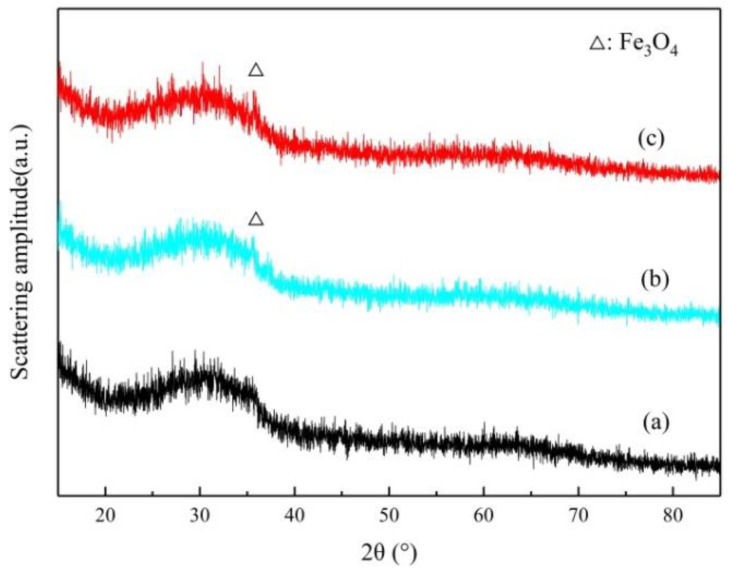
XRD patterns of the RGCS: (**a**) CSA0, (**b**) CSA05, and (**c**) CSA10.

As shown by the analysis in Figure 2, only diffuse peaks in the XRD patterns of the three samples can be seen. The diffraction patterns are in the shape of steamed bread, without sharp peaks. These findings are highly similar to the observations of other researchers [32,33] of amorphous structures, further indicating that the RGCS samples are mainly composed of amorphous minerals. By comparing the diffraction pattern of each RGCS sample to that of CSA0, a weak magnetite diffraction peak is identified in both CSA05 and CSA10 around 2θ = 35°, which indicates the formation of magnetite. As a consequence, the adding of Al_2_O_3_ may weaken the binding force between FeO and SiO_2_ in the amorphous structure and increase the activity of FeO, thus promoting the precipitation and growth of magnetite. In conclusion, RGCS exhibited high pozzolanic activity and an amorphous structure, which can spontaneously transform to a stable crystal structure under certain thermodynamic conditions.

### 3.3. Isothermal Calorimetry 

**Figure 3:**
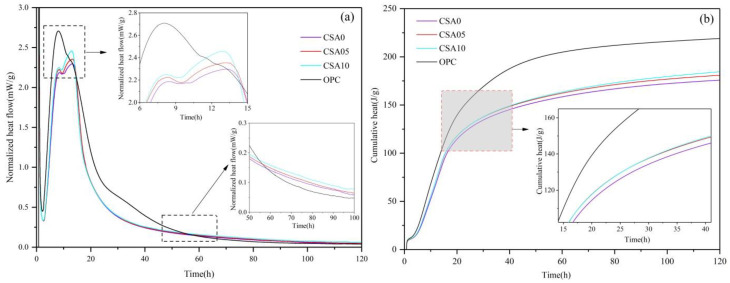
Normalized heat flow (**a**) and cumulative heat (**b**) measured at 25 °C by isothermal calorimetry for the OPC and blended cements paste.

The normalized heat flow curves (Figure 3a) and cumulative heat flow curves (Figure 3b) of OPC, CSA0, CSA05, and CSA10, as measured by isothermal calorimetry, are obtained. The characteristic values of heat flow and cumulative heat of the four samples are summarized in Table 2. In Figure 3a, the first heat flow peak was generated within a few minutes after the OPC or blended cement was mixed with water (the initial period (I)), which was derived from the exothermic hydration of C_3_S [34]. After a short exothermic period, the sample entered the induction period (II), the duration of which depended primarily on the lattice defects of C_3_S and was about 7 h–8 h. The early hydration of C_3_S generally occurred at the activation point where it was most likely to react with water, also known as a lattice defect [35]. The early hydration reaction induced Ca^2+^ on the surface of C_3_S to be incorporated into the aqueous solution, forming a negatively charged “silicon rich layer”. Then, the “silicon-rich layer” absorbed Ca^2+^ from the aqueous solution, forming “electric double layers” that prevented further hydrolysis of C_3_S. The electric potential difference (ξ potential) between the “electric double layers” gradually decreased due to the release of Ca^2+^ by the slow hydrolysis of C_3_S. After the electric potential difference was decreased to the point where it was unlikely to resist the coulombic forces between the ions, the acceleration period (III) began with the precipitation of Ca(OH)_2_ crystals and a small amount of C-S-H on the surface of C_3_S. This coincided with the second heat flow peak at about 8 h for OPC and blended cement paste samples. Compared to OPC, since RGCS replaced part of the OPC in the blended cements and produced a dilution effect, the second heat flow peak of the blended cements was significantly lower and slightly delayed. On the other hand, the second heat flow peak of CSA10 not only appeared earlier than the other blended cements, but also had higher values. This indicated that the Ca^2+^ dissociation on the C_3_S surface was significantly inhibited by RGCS, which limited the early exothermic hydration of C_3_S, but was effectively alleviated by the incorporation of Al_2_O_3_.

Interestingly, the hydration reaction of the blended cement samples proceeded up to about 13 h with a third heat flow peak that prolonged their acceleration period, but this signal was not captured in OPC. Comparing the third heat flow peak of the three blended cement samples, the exothermic peaks of CSA10, CSA05, and CSA0 were 2.48, 2.35, and 2.30 mW·g^−1^, respectively, and their values and durations were significantly higher than not only the second heat flow peak, but also the OPC heat flow value at the same period. Significantly, the heat release of the blended cement paste in the early stage of hydration was derived from the hydrolytic of C_3_S. However, in the late stage of acceleration period, the accumulation of hydration products on the surface of active particles (including but not exclusively C_3_S) hindered the hydration reaction. So, the unexpected appearance of the third peak in blended cement heat flow signal was mostly derived from the heat release of the pozzolanic reaction of active components on the surface of RGCS with CH. Additionally, the heat release was larger than that of the OPC hydration at the same time. In addition, the third heat flow peak was positively correlated with the adding amount of Al_2_O_3_ in RGCS. It was illustrated that the addition of Al_2_O_3_ increased the concentration of OH^−^ and Al^3+^ in the hydration system of blended cement and played a positive role in prolonging the acceleration period and promoting the dissociation of RGCS surface active ingredients.

During the deceleration period (IV), numerous hydration products were generated and covered the surface of C_3_S or RGCS, which lessened the exothermic rate of hydration. Then, it entered the stabilization period (V) after the exothermic rate of hydration decreased to near zero. However, during the stabilization period (starting from 55 h), the exothermic rates of the blended cement samples were all higher than that of the OPC. This suggested that the heat release of pozzolanic reaction was slightly higher than that of cement hydration reaction at hydration middle and later stage. Additionally, the normalized heat value curve of CSA10 was at the upper most, which demonstrated that the adding increase in Al_2_O_3_ contributed to the heat release of pozzolanic reaction at hydration later period.

Given in Table 2 and Figure 2b, the cumulative heat curves of the blended cement samples exhibited the identical trend but with lower values than OPC. There was no dispute that cement hydration reaction was the primary source of heat release from OPC and blended cement slurries. Additionally, the cumulative heat in 120 h of blended cement were 184.2 J/g, 180.7 J/g, and 175.6 J/g, respectively, significantly higher than 70% of the OPC (153.3 J/g). Apparently, the additional heat release was derived from the pozzolanic reaction of the dissociated active molecules on the RGCS surface with CH. Furthermore, the cumulative heat of the blended cements increased markedly with the increase in Al_2_O_3_ incorporation in RGCS. 

### 3.4. UCS Analysis

**Figure 4:**
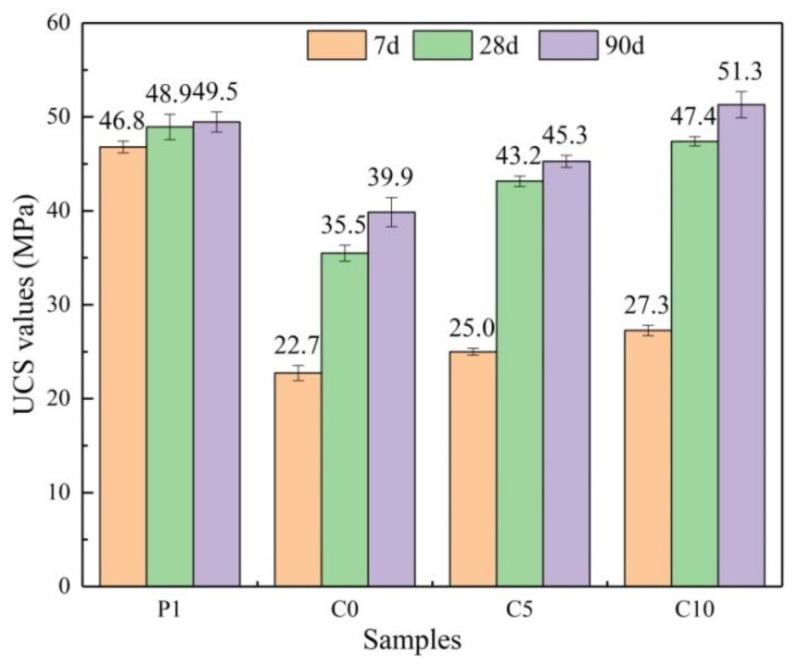
UCS values of mortars prepared using OPC and blended cement at 7 d, 28 d, and 90 d.

Given in Figure 4, the UCS values of C0, C5, and C10 (22.7, 25.0, and 27.3 MPa, respectively) reached only about 50% of the P1 UCS value (46.8 MPa) by the early stage of consolidation (7 d). This indicated that, in the early stage of the consolidation of blended cement mortars, the cement hydration reaction was dominant, and the pozzolanic reaction was not prominent. Further, the specific surface area of RGCS was larger than that of OPC [36], which made the finer RGCS particles encapsulate OPC particles, resulting in the insufficient diffusion exchange rate of cement particles and thus inhibiting the hydration reaction in the early stage. The UCS values of C5 and C10 were 10.1% and 20.3% higher, respectively, than C0, which indicated that increasing the content of Al_2_O_3_ in RGCS could effectively alleviate the lag in the early strength development of blended cement mortar.

As demonstrated in Table 3, the UCS development rate of P1 (only 0.1 MPa·d^−1^) showed a significant decreasing trend, indicating that the hydration reaction of OPC was largely completed within 7 d. In inclusion, the UCS values of blended cement mortar samples reached 35.5–47.4 MPa at 28 d, the UCS ratios ranged from 72.6% to 96.9%, and the UCS development rates varied from 0.61 to 0.96 MPa/d. The hydrolysis process of vitreous RGCS in blended cement mortar samples was completed within 7 d–28 d of these dates. Additionally, the UCS value of C10 was 26.9% higher than the standard strength, C0 was only 2.6%. This supports that the higher medium-term UCS growth depends on the high dose of Al_2_O_3_ in RGCS. After 90 d of hydration, the UCS development rate of all samples remained at 0.01–0.07 MPa/d with a small increasing trend, indicating that the hydration reaction was largely completed within 28 d. In summary, this suggests that Al_2_O_3_ plays a vigorous role in the UCS development of blended cement mortar throughout its life cycle, and mainly in the middle stage (7 d–28 d).

### 3.5. XRD Analysis of RGCS

**Figure 5:**
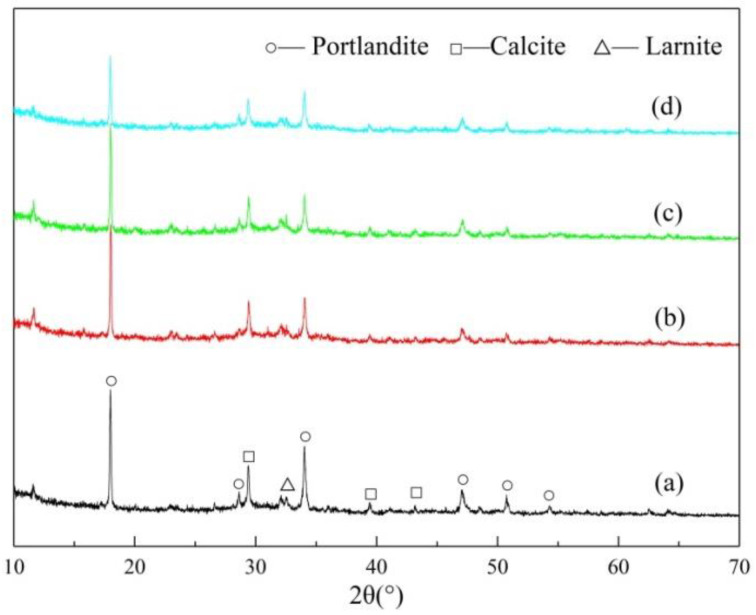
XRD patterns of the paste prepared using OPC and blended cements cured for 28 d: (**a**) OPC, (**b**) CSA0, (**c**) CSA05, and (**d**) CSA10.

XRD patterns of the pastes prepared using OPC and blended cements cured for 28 d are presented in Figure 5. Additionally, it shows that the samples had similar diffraction peaks at the same position. The main hydrated crystalline minerals of the samples were portlandite (CH), calcite (CaCO_3_), and larnite (β-C_2_S). Notably, it was difficult to detect C-S-H gel using XRD because of its amorphous structure. Of these minerals, the diffraction peak intensity of CH was the highest, indicating that it accounted for the most among the hydration products. The existence of CaCO_3_ was mainly due to the oxidation of cement itself, and some proportion of CH was carbonized by CO_2_ during curing [37]. Additionally, the existence of β-C_2_S was mainly due to its slow hydration rate. Some observations [38] have found the surface of β-C_2_S to still be covered with only a small quantity of amorphous hydrated calcium silicate after tens of days. In addition, the modification effect of Al_2_O_3_ on RGCS can be intuitively judged by the CH consumption, since the formation of CH was accompanied by the whole hydration process of cement. The research [39] has showed that CH not only had the positive impact of providing an alkaline environment for the hydration reaction, but also had the negative effect of increasing the pore ratio and reducing the later strength of cement or blended cement.

Among the crystalline minerals, the CH peak intensity of OPC paste at θ = 18° was the highest, and the CH peak intensity of the CSA0 paste was about 65% (less than 70%) of that of OPC. This clearly demonstrates that the active molecules released and reacted with CH, after the dissolution of GCS glass particles, resulting in partial consumption of CH. With the increase in the Al_2_O_3_ content in RGCS, the CH peak intensity decreased significantly. This showed that more active molecules were released from the dissociation of the RGCS surface with the higher Al_2_O_3_ content, which promoted the pozzolanic activity. The amount of carbonized CH needed to be excluded for the carbonization by CO_2_ during the samples curing in the quantitative analysis is noteworthy. Therefore, the content of CaCO_3_ from CO_2_ carbonization was calculated by subtracting the original content in the samples so as to obtain the quantity of carbonized CH. Additionally, TGA was usually used to quantitatively describe the change in CH content in the hydration products.

### 3.6. Thermogravimetric Analysis 

**Figure 6:**
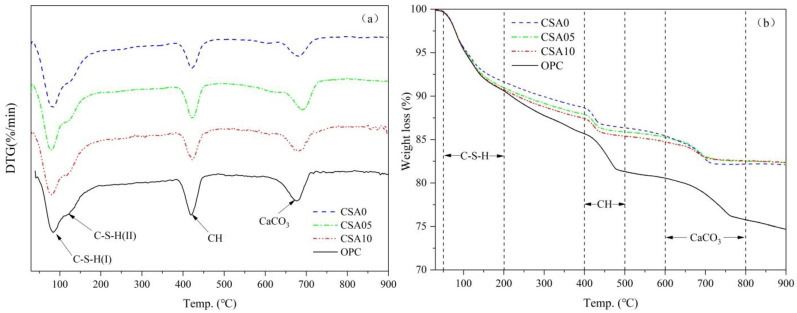
DTA/TGA curves of the pastes prepared using OPC and blended cements cured for 28 d: (**a**) DTA curves and (**b**) TGA curves.

The DTA/TGA curves of OPC and blended cement samples are shown in Figure 6. From the DTA curves, the first endothermic peak between 80 °C and 200 °C resulted from the separation of the C-S-H interlayer water [40]. Additionally, a weak signal on the right shoulder of the first peak (at about 130 °C) was consistent with the hypothesis proposed by Taylor [41], indicating the presence of two different structures of C-S-H. One was the tobermorite-type structure, or C-S-H (I). The other was the jennite-type structure with higher Ca/Si ratio, denoted C-S-H (II). Given in the TGA curves (Figure 6b), the addition of 30 wt% RGCS to the blended cement reduced the peak strength of C-S-H in CSA0 sample. Additionally, the consumption of CH in pozzolanic reaction accelerated the transformation of the C-S-H gel structure from C-S-H (I) to C-S-H (II). The improvement might benefit from the additional release of Ca^2+^ due to the decrease in OH^−^ by pozzolanic reaction, so that the improvement effect became increasingly obvious with the increase in Al_2_O_3_ in the RGCS. Regarding the quantity and structure of C-S-H gel, it is worth considering in the discussion that the largest amounts of C-S-H and C-S-H (II) are generated in the CSA10 sample.

Then, the other two endothermic peaks appeared at 400–500 °C and 600–800 °C, representing the decomposition of CH and decarburization of CaCO_3_, respectively. From TGA curves and the carbonization effect, the CH and CaCO_3_ contents in the system can been accurately obtained from Formula (1) [42,43]. So, the CH contents of OPC, CSA0, CSA05, and CSA10 were 26.06%, 14.93%, 12.87%, and 12.05% by TGA curves, respectively. Additionally, the fixed CH in CSA0, CSA05, and CSA10 samples were 18.1%, 29.5%, and 34.0% by calculation. It is noteworthy that the fixed CH of CSA05 increased by 11.4% compared to CSA0, but only by 4.5% for CSA10 compared to CSA05. The results showed that the largest value of fixed CH was obtained by pozzolanic reaction for CSA10 sample, and more active SiO_2_ or Al_2_O_3_ was released from RGCS. However, the expected increase in the fixed CH was not obtained with the addition of Al_2_O_3_ in RGCS.

Fixed CH (%) = [(CH_P_ × C%) − CH_G_]/(CH_P_ × C%) (1)

where CH_P_ and CH_G_ are the CH contents of the OPC and blended cement, respectively, and C% is the proportion of OPC in the sample, equal to 70%.

According to the irregular network theory proposed by Zachariasen [44], Al is usually used as an attacker of the Si-O covalent bond in glass, replacing the Si atom to form an ionic bond of low intensity, then forming a tetrahedral four-coordinate structure [AlO_4_] with the non-bridging oxygen (NBO). However, a network-forming body can also be used to form an octahedral hexacoordinate structure [AlO_6_] in the vitreous network when the bridging oxygen (BO) insufficient. The fixed CH as a quantitative characterization of RGCS pozzolanic activity has been discussed. Consequently, when the amount of Al_2_O_3_ added in RGCS was low (CSA05) and BO was sufficient, Al could destroy BO to form NBO as a network-changing body, which increased the disorder (entropy) in the RGCS glass system and enhanced its pozzolanic activity. However, when the amount of Al_2_O_3_ added in RGCS was high (CSA10) and BO was relatively insufficient, a proportion of the Al_2_O_3_ acted as a network-forming body, weakening the transformation from BO to NBO [45]. This is also why the expected fixed CH was not obtained with the addition of Al_2_O_3_ in RGCS.

### 3.7. FTIR Analysis

**Figure 7:**
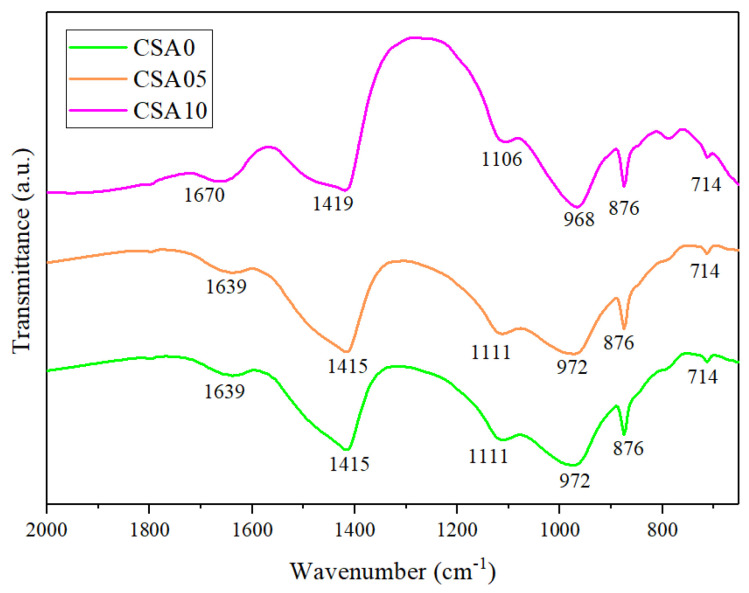
FTIR spectra of blended cement pastes at 28 days of curing.

Figure 7 shows the FTIR spectra for the blended cement pastes at curing ages of 28 days, and the similarities in FTIR spectra confirm a similar kind of reaction product formation for blended cement pastes. The bands near 1419 and 1415 are assigned to the asymmetric stretching vibration of C-O bonds of calcite [46]. The available peaks at 876 and 714 cm^−1^ are due to the bending vibration of C-O bonds in CO_3_^2−^ [47], which is attributed to the carbonation of CH by the pozzolanic reaction and the environment. The intensity of these peaks increases slightly with the addition of Al_2_O_3_, indicating a higher consumption of CH, resulting in a higher degrees of pozzolanic reaction. The absorption peaks at 1639 and 1670 cm^−1^ correspond to the bending vibration of the fundamental O-H bond in the water [48]. The peak at about 1111 cm^−1^ is due to the asymmetric stretching vibration of S-O bonds of SO_4_^2−^ groups in ettringite (Aft) [47], which has a small shift towards lower wavenumber and slightly deeper peak in CSA10 sample for providing more aluminates. The peaks at 972 and 968 cm^−1^ are due to the asymmetric stretching vibration of Si-O-Si or Si-O-Al bands in the SiO_4_ tetrahedron [49] The more intense peak can be observed for more C-S-H or C-A-S-H gel being produced with the addition of Al_2_O_3_ in RGCS. Additionally, the lower wavenumber is mainly due to the increase in Al/Si and Ca/Si ratios, resulting in two C-S-H structural differences. The FTIR results have verified our DTG/TGA measurements in Section 3.6, so that with the addition of Al_2_O_3_ in RGCS, the generation of C-S-H gels is promoted by accelerating the consumption of CH, while a higher proportion of Al facilitates the transformation of C-S-H (I) to C-S-H (II).

### 3.8. SEM Analysis 

**Figure 8:**
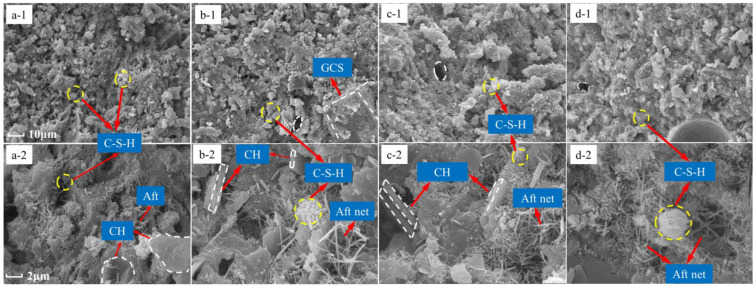
SEM micrographs of the OPC and blended cement pastes after 28 d of curing: (**a-1**) OPC (×1000), (**a-2**) PC (×5000), (**b-1**) CSA0 (×1000), (**b-2**) CSA0 (×5000), (**c-1**) CSA05 (×1000), (**c-2**) CSA05 (×5000), (**d-1**) CSA10 (×1000), (**d-2**) CSA10 (×5000).

To further characterize the pozzolanic activity of RGCS, the morphology and quantity of hydration products, the size and distribution of the pore, and the microstructure compactness were qualitatively examined by SEM analysis of OPC and blended cement paste samples cured for 28 d. Given in Figure 8, the SEM spectra of OPC, CSA0, CSA05, and CSA10 paste samples at magnifications of 1000 and 5000 were shown, respectively. Both OPC and blended cement samples presented sufficiently dense microstructures but higher compactness for OPC. Additionally, the pores of OPC sample had a small size with a dense distribution, but were larger and slightly dispersed for blended cement samples. This difference may result from the fact that 30 wt% OPC was replaced in blended cement, and fewer hydration products were generated. By observing the morphology and quantity of hydration products, lots of fibrous and flocculent C-S-H gels in the OPC sample (Figure 8(a-1,a-2) clung to the poorly crystallized area. A small quantity of Aft was randomly attached to the surface of hexagonal plate-like CH. It illustrates that the presence of a large number of CH crystals in the OPC sample maintains the alkalinity of the system at a high level.

In the CSA0 sample (Figure 8(b-1,b-2)), the morphology of C-S-H was largely consistent with that of OPC, but the amount of hydration products was significantly reduced. Notably, a large GCS particle was highlighted in the structure, and the exposed surface was “etched” by a small quantity of hydration products. Many CH crystals were staggered to form a blank area, which was structurally weak. Moreover, a large number of needle-like Aft grew and overlapped with each other to form a local area network in the staggered CH blank area. Therefore, the partial substitution of RGCS for OPC leads not merely to the decay of hydration products but also to the weakening of the microstructure.

Furthermore, a small quantity of hydration products attached to the surface of the GCS proved the existence of the pozzolanic activity, but the existence of more CH crystals indicated that this pozzolanic activity was low and consumed only a limited portion of the CH. Further, the small amount of hydration products adhering to the surface of GCS and the fine consumption of CH crystals demonstrated the low pozzolanic activity in the CSA0 sample, thus limiting the hydration reaction. However, the CH crystals gradually decreased by the erosion of the pozzolanic reaction with the increase in Al_2_O_3_ content in the RGCS (Figure 8(c-1,c-2)). The additional hydration products, such as C-S-H and Aft, filled the staggered blank area of CH or covered the surface of unreacted GCS. From Figure 8(d-1,d-2), the compactness of the CSA10 sample structure was better than that of OPC, but the increase in Aft may have a negative effect on the growth of CSA10 strength.

**Figure 9:**
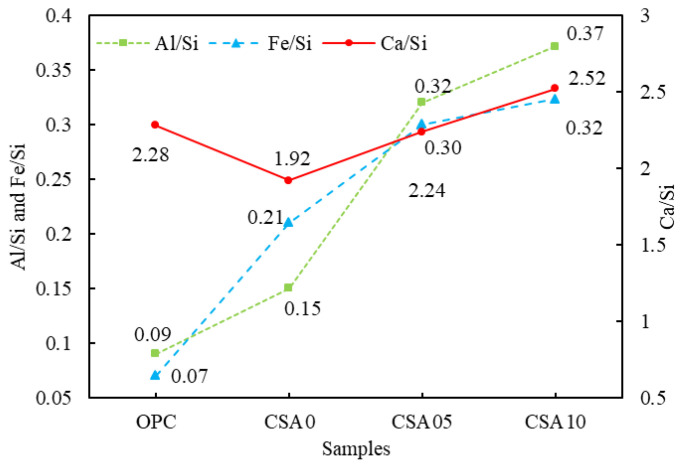
Averaged values of atomic ratios for the C-S-H gel formed in the OPC and blended cement pastes after 28 d of curing.

For further analysis of the differences in C-S-H gel compositions of OPC and blended cement samples, semi-quantitative analysis of Ca/Si, Al/Si, and Fe/Si ratios in EDS was conducted using the random average method. The results, displayed in Figure 9, showed that the Ca/Si ratios of CSA0 (1.92) and CSA05 (2.24) samples were lower than that of OPC (2.28), but the Ca/Si ratio of CSA10 (2.52) was higher than that of OPC. This supports that with the increase in Al_2_O_3_ content in RGCS, Al replaces Ca and plays the role of network-changing body to enhance the pozzolanic activity. Then, the dissociated Ca participated in the pozzolanic activity to form a higher Ca/Si ratio C-S-H. Meanwhile, the Al/Si and Fe/Si ratios in blended cement samples were much larger than those in the OPC and were proportional to the content of Al_2_O_3_ in the RGCS. The data indicate that RGCS released active components such as Al and Fe during dissociation and formed C-S-H with high Al/Si and Fe/Si ratios by pozzolanic reaction. Notably, the increase in Al_2_O_3_ adding in RGCS can effectively promote this process.

## 4. Conclusions

This study explored the feasibility of RGCS as a SCM, which was obtained by adding Al_2_O_3_ into liquid CS. The following findings were concluded.

1. In terms of physicochemical properties and mineral-phase composition, RGCS with the addition of Al_2_O_3_ leads to an increase in magnetite precipitation by weakening the bond between FeO and SiO_2_ and exhibits a highly amorphous structure. However, the BET surface areas of CSA0, CSA05, and CSA10 are 0.67, 0.67, and 0.69 m^2^·g^−1^, respectively, showing negligible variations.
2. The cement and GCS mixture show increases in exothermic rate and cumulative heat release at early-age hydration after the modification using Al_2_O_3_, which can accelerate the pozzolanic reaction. The sample of CSA10 exhibits the most violent reaction with the second peak value of 2.25 mW·g^−1^ and the 120 h cumulative heat of 184.2 J·g^−1^.
3. From the UCS testing of blended cement and OPC mortars, C10 gains higher UCS ratios and UCS development rate from 7 d to 28 d than that of C0 and C05, and a higher UCS value (51.3 MPa) than OPC (49.5 MPa) at 90 d of curing. This indicates that the UCS development of C10 mortar is more advantageous than OPC at the middle and later curing stage (7–90 d).
4. Regarding TGA, the fixed CH of the cured 28-day blended cement slurry is 18.1%, 29.5%, and 34.0% for CSA0, CSA05, and CSA10 slurries, respectively. The CSA10 slurry exhibits the highest pozzolanic activity, but the expected increase in the fixed CH was not obtained with the addition of Al_2_O_3_ in RGCS. This is attributed to the fact that the transformation from BO to NBO is weakened, and part of Al_2_O_3_ plays the role of network-forming body when the content of Al_2_O_3_ is over 5 wt% in RGCS. Additionally, the FTIR results verify DTG/TGA measurements.
5. From SEM and EDS, the addition of Al_2_O_3_ accelerates the dissociation of RGCS, thus releasing more active molecules, enhancing the growth of hydration products of blended cement samples, filling the structurally weak regions, promoting the formation of C-S-H with high Ca/Si, Al/Si, and Fe/Si ratios, and reaching the optimum level at 10 wt% addition of Al_2_O_3_.

## Figures and Tables

**Table 1:** Physicochemical properties of the RGCS and OPC.

Constituents	Chemical Composition (% by Mass)			
	CSA0	CSA05	CSA10	PC
FeO	35.89	33.91	32.15	
SiO_2_	33.40	32.70	32.70	18.10
CaO	4.00	3.90	3.70	62.10
Fe_2_O_3_	7.14	9.11	9.47	2.80
Al_2_O_3_	3.50	6.60	9.80	4.90
MgO	1.39	0.82	0.82	1.20
ZnO	1.35	1.02	0.95	
Cu_2_O	1.65	0.68	0.64	
Cr_2_O_3_	0.85	0.27	0.26	
Sb_2_O_5_	0.11	0.04	0.04	
Pb_2_O_3_	0.04	0.01	0.01	
Physical characteristics				
BET surface area (m^2^·g^−1^)	0.67	0.67	0.69	0.47
Density (g·cm^−3^)	3.56	3.49	3.46	3.08

**Table 2:** Characteristic values of normalized heat flow and cumulative heat.

Sample	The Second Peak		The Third Peak		Cumulative Heat (J/g)		
	Time of Peak Occurrence (h)	Peak Value (mW/g)	Time of Peak Occurrence (h)	Peak Value (mW/g)	20 h	60 h	120 h
OPC	8.0	2.72	–	–	139.4	204.8	219.0
CSA0	8.4	2.20	13.2	2.30	113.5	158.0	175.6
CSA05	8.4	2.23	13.0	2.35	117.1	162.0	180.7
CSA10	8.2	2.25	12.8	2.48	117.3	163.4	184.2

**Table 3:** The UCS ratio and UCS development rate for all tested mortars.

Sample	UCS Ratio (%)			UCS Development Rate (MPa·d^−1^)	
	7 d	28 d	90 d	from 7 d to 28 d	from 28 d to 90 d
P1	100.0	100.0	100.0	0.10	0.01
C0	48.6	72.6	80.6	0.61	0.07
C5	53.4	88.3	91.5	0.87	0.03
C10	58.2	96.9	103.7	0.96	0.06

## Data Availability

All data in this paper are obtained from our experiment and are authentic and reliable. The publication of data has obtained the consent of all authors.
